# Healing of Vertebral Compression Fractures in the Elderly after Percutaneous Vertebroplasty—An Analysis of New Bone Formation and Sagittal Alignment in a 3-Year Follow-Up

**DOI:** 10.3390/jcm11030708

**Published:** 2022-01-28

**Authors:** Yuh-Ruey Kuo, Ting-An Cheng, Po-Hsin Chou, Yuan-Fu Liu, Chao-Jui Chang, Cheng-Feng Chuang, Pei-Fang Su, Ruey-Mo Lin, Cheng-Li Lin

**Affiliations:** 1 Department of Orthopedic Surgery, National Cheng Kung University Hospital, Tainan City 704, Taiwan; raykuo19@gmail.com (Y.-R.K.); celeblack1030@gmail.com (T.-A.C.); vx9527@hotmail.com (C.-J.C.); 2 School of Medicine, National Yang Ming Chiao Tung University, Taipei 112, Taiwan; choupohsin@gmail.com; 3 Department of Orthopedics and Traumatology, Taipei Veterans General Hospital, Taipei 112, Taiwan; 4 Department of Orthopedic Surgery, National Cheng Kung University Hospital Douliu Branch, Douliu City 640, Taiwan; andwantfly@hotmail.com; 5 Department of Statistics, National Cheng Kung University, Tainan City 701, Taiwan; i2mshy@gmail.com (C.-F.C.); pfsu@ncku.edu.tw (P.-F.S.); 6 Department of Orthopedic Surgery, An-Nan Hospital, China Medical University, Tainan City 709, Taiwan; D71081@mail.tmanh.org.tw; 7 Department of Orthopedics, College of Medicine, National Cheng Kung University, Tainan City 701, Taiwan; 8 Musculoskeletal Research Center, Innovation Headquarter, National Cheng Kung University, Tainan City 701, Taiwan; 9 Medical Device Innovation Center (MDIC), National Cheng Kung University, Tainan City 701, Taiwan

**Keywords:** vertebroplasty, osteoporotic vertebral compression fractures, new bone formation, restoration, spine

## Abstract

Background: Vertebral compression fractures, resulting in significant pain and disability, commonly occur in elderly osteoporotic patients. However, the current literature lacks long-term follow-up information related to image parameters and bone formation following vertebroplasty. Purpose: To evaluate new bone formation after vertebroplasty and the long-term effect of vertebroplasty. Methods: A total of 157 patients with new osteoporotic compression fractures who underwent vertebroplasty were retrospectively analyzed. The image parameters, including wedge angles, compression ratios, global alignment, and new bone formation, were recorded before and after vertebroplasty up to three years postoperatively. Results: The wedge angle improved and was maintained for 12 months. The compression ratios also improved but gradually deteriorated during the follow-up period. New bone formation was found in 40% of the patients at 36 months, and the multivariate analysis showed that this might have been related to the correction of the anterior compression ratio. Conclusions: Vertebroplasty significantly restored the wedge angles and compression ratios up to one year postoperatively, and new bone formation was noted on plain radiographs, which increased over time. Last, the restoration of vertebral parameters may contribute to new bone formation.

## 1. Introduction

Vertebral compression fractures (VCFs) commonly occur in the elderly, especially among women, and are typically associated with osteoporosis [1,2]. They may lead to back pain, the inability to carry out the activities of daily living, and deterioration of quality of life. Hence, it is of great importance to obtain effective treatment for senile patients with VCFs. Currently, various treatments are available, including conservative treatments such as pain control, bed rest, bracing, and physical therapy. In addition, minimally invasive procedures such as percutaneous vertebroplasty (PVP) or percutaneous kyphoplasty (PKP) with cement augmentation of the fractured vertebrae are also widely used to relieve disabling pain [3,4,5]. However, the therapeutic effects of these interventions still remain debatable.

In 2015, in an article published in the Cochrane Review, Buchbinder et al. revealed that there are no demonstrable clinically important benefits of PVP for osteoporotic VCFs compared with a sham procedure [6]. Boszczyk et al. also reported that PVP provides no clinically significant benefits over a placebo [7]. On the contrary, numerous studies have stated that PVP is a better choice over non-operative treatments in terms of pain alleviation, improvements in daily functions, and restoration of vertebral height, as well as the wedge angle of the fractured vertebrae [4,8,9,10,11]. However, most of these studies lack long-term follow-up data more than 1 year postoperatively. Therefore, there is still insufficient evidence in the literature as to whether or not PVP can be sustained for a longer time span.

In the healing process after a fracture, it is considered to be stabilized and healed when bony union is achieved. Nevertheless, the union status of fractured vertebrae after PVP has not been thoroughly addressed. Although multiple studies have focused on evaluating the effects of vertebral cement augmentation, such as height restoration and wedge angle correction, few studies have evaluated bone-healing after PVP. Kawaguchi et al. first reported that bone emerging between the fractured vertebra and the adjacent vertebrae was present in 14 patients after PVP and further developed to a solidified form during follow-ups [12]. They indicated that PVP induces segmental bony spur and callus formation and thus possibly plays a role in converting an atrophic to a hypertrophic vertebral pseudoarthrosis by serving as a mechanical stabilizer. Braunstein et al. also detected large amounts of newly formed callus surrounding the injected cement in a postmortem cadaveric spine, indicating that the new bone around the fractured vertebrae is present after PVP [13].

Owing to the limited number of studies on the bone-healing capability and long-term effects of PVP on senile vertebral compression fractures, this study was aimed toward evaluating new bone formation, spinal alignment, and vertebral height restoration after PVP in a 3-year follow-up. We hypothesized that fracture healing would occur 1 year after PVP for VCF. The spinal alignment and vertebral height restoration would also be noted postoperatively.

## 2. Materials and Methods

### 2.1. Subjects

This was a retrospective cohort study including 678 cases of PVP in a tertiary medical center between April 2013 and December 2016 that were reviewed based on medical records and radiographic images. Patients who underwent single-level vertebroplasty due to recent compression fractures based on image reports of bone scan, computed tomographic scan, or magnetic resonance scan after failed conservative medical therapy, defined as either having intractable back pain at 4–6 weeks after initiation of fracture care or intolerable severe back pain at 2 weeks after initiation of fracture care were included. Patients with concurrent adjacent fractures, previous spinal intervention (instrumentation, decompression or fusion) on the targeted or adjacent levels, pathological fractures, and revision or re-do surgeries were excluded. We also analyzed 57 patients with single-level compression fractures who did not undergo PVP for comparison. These patients were treated conservatively with analgesics and orthoses. As for the surgical technique, patients were placed prone on a Wilson frame, allowing for the potential reduction of the collapsed wedge angle by this position. Demographic data were recorded, including age, sex, T-score, symptoms, fracture level, the presence of the intravertebral vacuum clef sign, cement type, cement amount, and if present, and the reason for subsequent re-operation or re-vertebroplasty. This study was approved by our institutional Review Board (IRB number: B-ER-107-312).

### 2.2. Measurement of Vertebral Parameters and New Bone Formation

**Figure 1:**
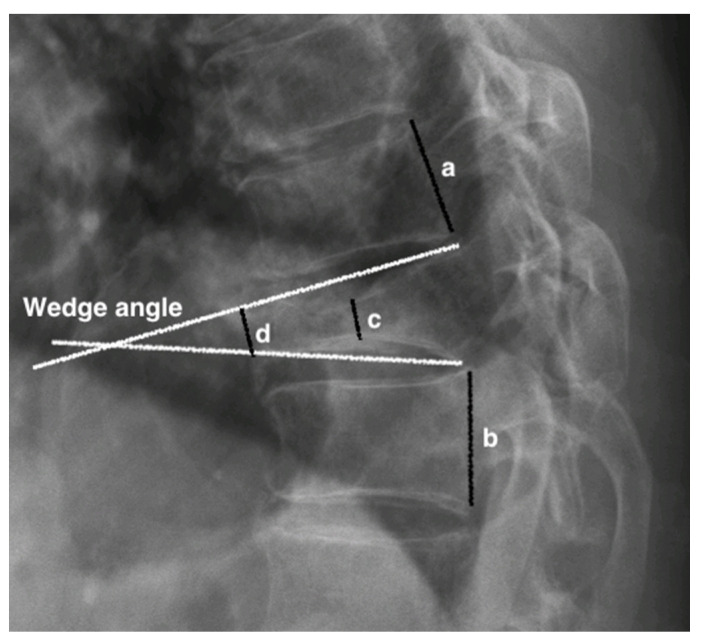
The measurement of radiographic parameters. The wedge angle is defined as the angle between the superior and inferior endplates of the collapsed vertebra. c and d represent the anterior and middle vertebral height, respectively. The normal vertebral height was defined as (a + b)/2. The anterior and middle compression ratios (ACR and MCR) were calculated as [1 − (ant or mid-height/normal height)] × 100%.

**Figure 2:**
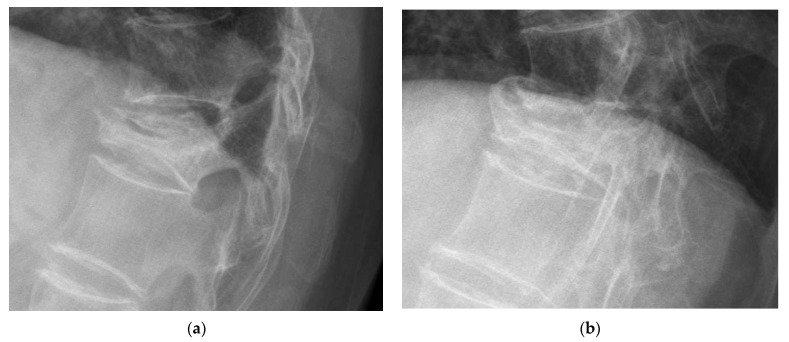
New bone/callus formation. (**a**) Vertebral compression fracture with the vacuum phenomenon. (**b**) Bone formation emerging between the affected vertebra and the adjacent vertebrae.

Plain film radiographs were examined at various time points: preoperatively, immediately postoperatively, 1 month postoperatively (PO), 6 months PO, 12 months PO, 24 months PO, and 36 months PO. Radiographic assessments were completed by two individual observers. Inter- and intra-rater Interclass Correlation Coefficients for Reliability (ICC) were calculated, which were 0.864 and 0.930, respectively. Vertebral parameters and spinal alignments were measured at each time point. The vertebral parameters (Figure 1) included: (1) the wedge angle, which was defined as the angle between the line drawn from the superior and inferior endplates of the collapsed vertebra, (2) the anterior and middle vertebral height (d and c, respectively), and (3) the anterior and middle compression rate (ACR and MCR, respectively), which was defined as [1 − (anterior height or middle height/normal height)] × 100%, where normal height was the average height measured from the posterior superior endplate and the inferior endplate of each adjacent vertebral body (a and b). To determine spinal alignment, we measured the thoracic kyphotic angle or the lumbar lordotic angle. The thoracic kyphotic angle was defined as the angle between the T4 superior endplate and the T12 inferior endplate. The lumbar lordotic angle was defined as the L1 superior endplate and the S1 superior endplate. The presence of new bone/callus formation was defined as bone emerging between the affected vertebra and the adjacent vertebrae based on plain anteroposterior and lateral radiographs (Figure 2) [12].

### 2.3. Statistical Analysis

The data analysis included both descriptive and inferential statistics. Descriptive statistics were presented as estimated means (standard deviations, SD) for the continuous variables and as frequencies for the categorical variables. The Wilcoxon rank-sum test, as well as a chi-squared test, were used to evaluate the demographic data and the preoperative and postoperative parameters. A Kaplan–Meier survival curve was used for the purpose of comparing the serial changes in the percentage of positive new bone formation. A generalized estimating equations (GEE) analysis was conducted to compare serial postoperative image parameters with preoperative image parameters (i.e., the wedge angle, the compression ratios, lumbar lordosis, and thoracic kyphosis). All statistical tests were 2-sided, and a *p*-value less than 0.05 was considered to indicate statistical significance. All analyses were performed using statistical software R 3.3.1 for Windows (https://www.r-project.org, accessed on 28 December 2021) and SPSS software (version 17.0; SPSS, Chicago, IL, USA).

## 3. Results

A total of 157 patients met the inclusion criteria, including 38 males and 119 females. The mean age at the intervention was 75.2 years old, and the average T-score was −2.76 (Table 1).

**Figure 3:**
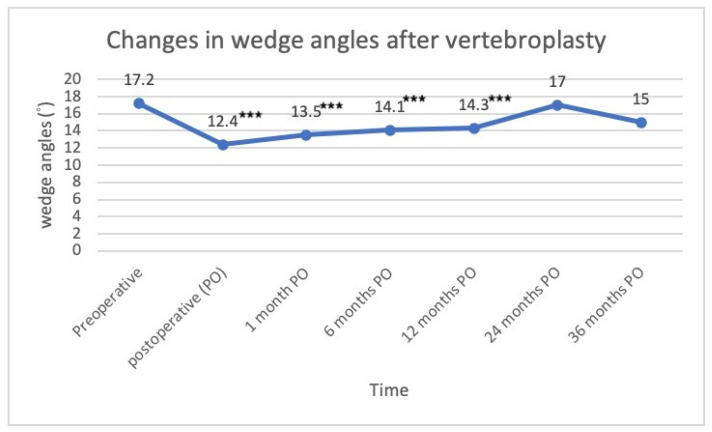
Changes in the wedge angles following vertebroplasty (*** *p*-value < 0.001, which was compared with preoperative value).

**Figure 4:**
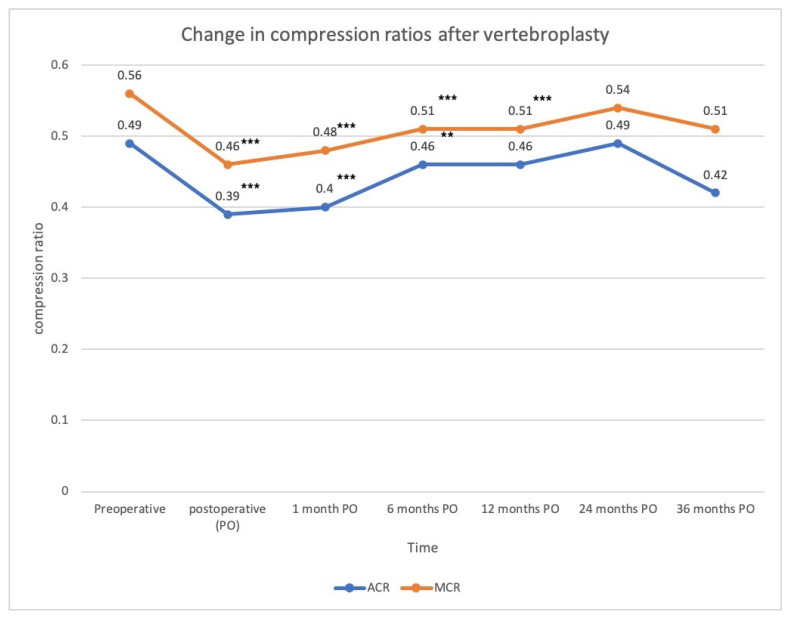
Change in anterior (ACR) and middle (MCR) compression ratios following vertebroplasty (*** *p*-value < 0.001, ** *p*-value < 0.01, which were compared with preoperative value).

The serial changes in the wedge angles were as follows: pre-op: 17.2°, immediately post-op: 12.4°, post-op 1 month (1 m): 13.5°, post-op 6 months (6 m): 14.1°, post-op 12 months (12 m): 14.3°, post-op 24 months (12 m): 17°, and post-op 36 months (36 m): 15°. Compared with the pre-op values, there was a statistically significant difference until post-op 12 m (*p* < 0.05) (Figure 3). The serial changes in the anterior vertebral compression ratio were as follows: pre-op: 49%; immediately post-op: 39%; post-op 1 m: 40%; post-op 6 m: 46%; post-op 12 m: 46%; post-op: 24 m 49%; post-op: 36 m 42%. Compared with the pre-op values, a statistically significant difference was reached immediately post-op, at post-op 1 m and 6m (*p* < 0.05). The middle vertebral compression ratios were as follows: pre-op: 56%; immediately post-op: 46%; post-op 1 m: 48%; post-op 6 m: 51%; post-op 12 m: 51%; post-op 24 m: 54%; post-op 36 m: 51%. Compared with pre-op values, immediately post-op, at 1 m, 6 m, and 12 m post-operatively, there was a statistically significant difference (*p* < 0.05), but there were no differences at the 24 m and 36 m follow-up (Figure 4).

**Figure 5:**
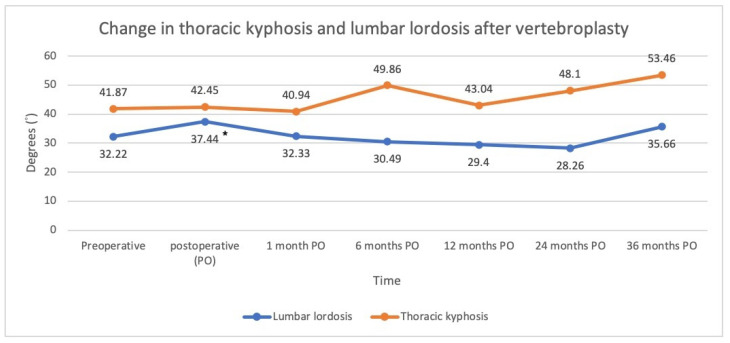
Changes in spinal alignment following vertebroplasty (* *p*-value < 0.05, which were compared with preoperative value).

The thoracic kyphotic angles were as follows: pre-op: 41.87°; immediately post-op: 42.45°; post-op 1 m: 40.94°; post-op 6 m: 49.86°; post-op 12 m: 43.04°; post-op 24 m: 48.1°; post-op: 36 m 53.46°. Compared with the pre-op values, there was no statistical significance at any time point. The lumbar lordotic angles were as follows: pre-op: 32.22°; immediately post-op: 37.44°; post-op 1 m: 32.33°; post-op 6 m: 30.49°; post-op 12 m: 29.4°; post-op 24 m: 28.26°; post-op 36 m: 35.66°. Compared with the pre-op values, there was statistical significance only in the immediate post-op value (*p* < 0.05) (Figure 5).

**Figure 6:**
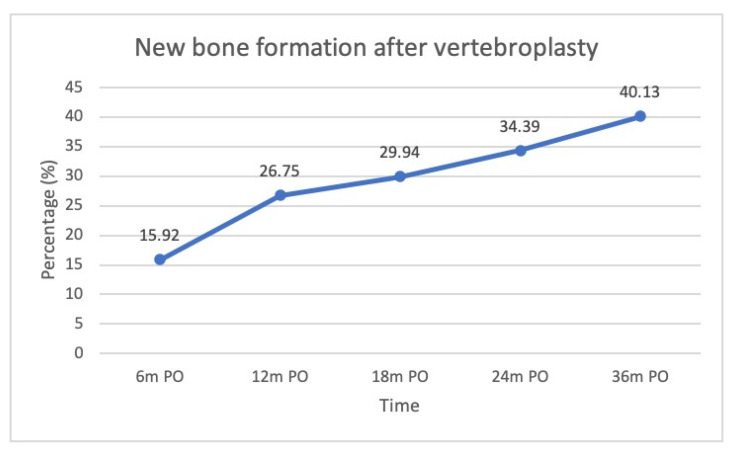
The percentage of new bone formation following vertebroplasty.

**Figure 7:**
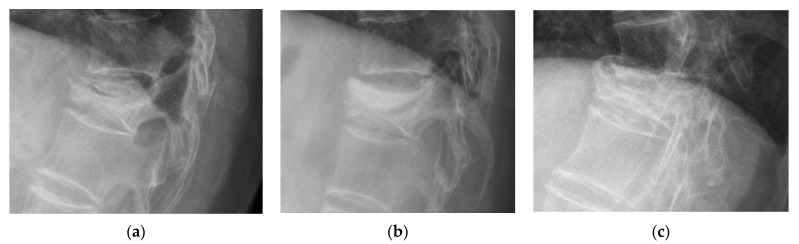
Demonstrated case of new bone formation. (**a**) A case of a T12 compression fracture with a vacuum sign. (**b**) Immediate postoperative lateral film after PVP. (**c**) Callus formation observed postoperatively at 12 months.

**Figure 8:**
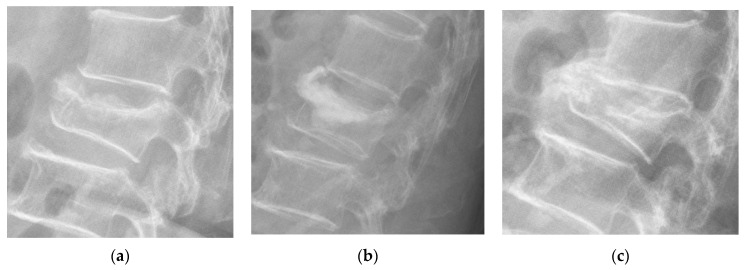
Demonstrated case of new bone formation. (**a**) A case of an L2 compression fracture. (**b**) Immediate postoperative lateral film after PVP. (**c**) Callus formation observed postoperatively at 24 months.

New bone formation was identified in 16% of the patients at post-op 6 months, 27% at post-op 12 months, 30% at post-op 18 months, 34% at post-op 24 months, and 40% at post-op 36 months. Cases with new bone formation were also demonstrated (Figure 6, Figure 7 and Figure 8).

Comparing the two groups with or without new bone formation at 6 months postoperatively, the group with new bone formation had a higher amount of injected cement (*p* < 0.05). There were no statistically significant differences in age, the preoperative wedge angles, the preoperative compression ratios, the T-scores, thoracic kyphosis, lumbar lordosis, and the presence of vacuum signs. Nonetheless, the group with new bone formation at 6 months had a significantly higher wedge angle correction, and the group with new bone formation at 24 months had a significantly higher ACR correction (Table 2). At *p*-values < 0.1, the univariate analysis showed that the amount of cement, the presence of vacuum signs, ACR correction, thoracic kyphosis correction, and lumbar lordosis correction might contribute to new bone formation. The further multivariate analysis showed that the anterior compression ratio correction was associated with new bone formation (Table 3).

### Comparison with Conservatively Managed Group

Fifty-seven patients with single-level compression fractures but not undergoing PVP were treated conservatively with analgesics and orthoses. The presence of new bone formation was statistically higher in the PVP group at all time points (6 m, 12 m, 24 m and 36 m). As for the wedge angle, the conservative group had higher wedge angles compared with the PVP group, but statistical significance was only noticed in 6m PO. As for the ACR and MCR, there was only statistical significance in preoperative values, whereas those for the PVP group were significantly higher.

## 4. Discussion

Osteoporosis affects over 10,000,000 people worldwide and results in extensive public health and economic burdens. Vertebral compression fractures occur commonly in aging populations, with a reported annual incidence of 750,000 in the USA [14]. PVP has been considered to be one of the treatments that can be used for pain relief, improvements in quality of life, and restoration of vertebral height. The incidence of PVP was increasing in previous literature, and it could cause significant socioeconomic burdens such as hospitalization and insurance costs [14]. Therefore, it could be of importance to evaluate the long-term effects of PVP for cost-effectiveness concerns. We analyzed the long-term effects of vertebroplasty up to 3 years postoperatively. This might provide some values regarding decision-making and health education between patients with osteoporotic vertebral compression fractures and treating physicians [15]. New bone formation after PVP was observed in previous studies; however, it was only a small sample without a between-group comparison with and without new bone formation [12]. In the present study, the wedge angle improved after PVP and was maintained for 12 months. The anterior and middle vertebral compression ratio also improved after PVP but gradually deteriorated during the follow-up. The PVP did not have significant effects on the thoracic kyphotic and lumbar lordotic angles. In addition, new bone formation was found in 40% of the patients at the 36-month follow-up. The group with new bone formation had a significantly higher amount of cement injected. The multivariate analysis showed that ACR correction might contribute to the formation of new bone.

### 4.1. Trend in the Radiographic Parameters

The changes in the radiographic parameters in the present study improved significantly immediately after the PVP but gradually deteriorated during the serial follow-up. Several studies also reported similar trends in the wedge angle and vertebral height restoration within short term follow-up periods [16,17,18,19,20]. In the present study, significant improvements in the wedge angle were found after PVP immediately, and the effect was maintained up to postoperative 12 months. The anterior compression ratio and the middle compression ratio also improved significantly and were maintained for up to 12 months. Therefore, a long term clinical study of more than 1 year is warranted to understand the effects of PVP on the VCF after 1 year.

Although different studies cannot be compared directly, the findings of the present study were generally consistent with those in the literature. The present study revealed that wedge angle significantly improved from 17.1° to 12.4° after PVP. The improvements in the wedge angle (4.7°) in the present study were similar to those found in previous studies, ranging from 1.5° to 7.1° [16,17,18,20,21,22,23,24,25,26]. Liu et al. found a significant reduction in the wedge angle from 15.5° to 12.2° in 50 patients who underwent PVP, and Teng et al. also reported that the wedge angle improved 7.1° postoperatively [16,24]. Ee and Martikos et al. both found a decrease in the wedge angle by 2.7° and 1.5° at mean follow-up periods of 24 months and 2.8 years, respectively [10,18]. Griffoni et al. also found improvements in the wedge angle from 10.8° to 8.8° at a final follow-up at 1 year postoperatively [12]. Although these studies showed significant improvements in the wedge angle, they only compared the preoperative and postoperative results, and no serial change was recorded during their follow-up periods. Garnier et al. found that wedge angle improved from 12° to 10° immediately after PVP, yet increased slightly to 11° 3 months later [17]. In a study conducted by Schofer et al., the wedge angle decreased from 11.4° to 9.3° immediately after PVP but increased to 10.4° at the final follow-up of around 13.7 months [20]. Chen et al. reported that the wedge angle improved from 20.57° preoperatively to 16.03° immediately after the PVP, but the reduction gradually progressed to 16.11°, 16.26°, and 16.43° at the 1, 3, and 6-month postoperative follow-ups, respectively [16]. The findings of the present study revealed that the wedge angle significantly improved from 17.1° to 12.4° immediately after PVP, but gradually deteriorated to 13.5°, 14.1°, 14.3°, 17°, and 15° during the serial follow-up at 1, 6, 12, 24, and 36 postoperative months, respectively.

In terms of vertebral height restoration, the compression ratio improved significantly from 49% to 39% for the anterior compression ratio and from 56% to 46% for the middle compression ratio. The improvement in the compression ratio (10%) in the present study was also ranged from 3.56% to 16.7%, as reported previously [16,19,25,26,27]. Teng and Yan et al. revealed improvements in the anterior height from 48.5% to 65.2% and from 53.4% to 64.5% after PVP with the measurement based on the posterior height of the adjacent normal vertebrae [25,27]. Chen et al. showed an increased restoration percentage for the vertebral height of 3.56% for the anterior vertebral height and 5.37% for the middle vertebral height [16]. However, these studies only compared data before and after PVP and lacked a serial follow-up. In Movrin’s study, the vertebral height at the most compressed area was measured and compared with the same site in the adjacent normal vertebrae [19]. The compression rate improved from 55.5% to 65% on the first postoperative day but slightly decreased to 63.5% at the 1-year follow-up. Chang et al. [26] prospectively analyzed 28 patients who underwent PVP and found that the vertebral height restoration rate increased from 74% to 79% at the final 2-year follow-up. The findings of the current study revealed that the compression ratio of the anterior vertebral height decreased significantly from 49% to 39% right after PVP, but gradually increased to 40%, 46%, 46%, 49%, then 42% at 1 month, 6 months, 1 year, 2 years, and 3 years, respectively. The middle compression ratio also improved from 56% to 46%, yet worsened to 48%, 51%, 51%, 54%, and then 51% during the serial 3-year follow-up. The anterior and middle vertebral compression ratio improved after PVP but gradually deteriorated during the follow-up.

The present study is one of the few studies evaluating changes in thoracic kyphosis and lumbar lordosis before and after vertebral augmentation for VCF. It has been reported that osteoporotic compression fractures worsen global kyphotic deformities not only locally but also globally, leading to sagittal imbalance [28]. Studies regarding the effects of vertebral augmentation on global alignment are emerging, many of which have shown the potential effect of restoring sagittal alignment. Yokoyama et al. reported that lumbar lordosis was significantly increased after kyphoplasty [29]. Cao et al. reported that lumbar lordosis was increased by 2 degrees after kyphoplasty and that compression fractures in the thoracolumbar region (T10-L2) achieved the better restoration of sagittal balance after the intervention [28]. Although there was improved alignment after kyphoplasty for VCF, our results showed that PVP did not have a significant effect on the thoracic kyphotic and lumbar lordotic angle postoperatively. Nevertheless, thoracic kyphosis increased as time went on and became significantly greater than the preoperative value at 3 postoperative years. The possible reason for this phenomenon might be the natural aging process or new VCF at other levels [30]. The findings of the present study suggested that single level PVP may have limited influence on the correction of sagittal alignment.

### 4.2. New Bone Formation

New bone formation was observed after the injection of cement into the collapsed vertebrae. Kawaguchi et al. reported that 28.5% of their patients had new bone formation, which they termed callus formation in their study, with a mean follow-up of 21 months [12]. They believed that segmental remodeling, such as callus formation after PVP, indicated that cement might serve as a mechanical stabilizer in the collapsed vertebrae. Similar to their findings, we found that new bone formation occurred in 34% of our patients at 24 months postoperatively. The present study further showed that the serial proportion of new bone formation increased gradually over time, with 16%, 27%, 34%, and 40% of our patients at 6 months, 1 year, 2 years, and 3 years, respectively.

### 4.3. Comparison between Groups with and without New Bone Formation

In the present study, there was no significant difference observed between the preoperative parameters in the groups with and without new bone formation, including the preoperative wedge angles, compression ratios, thoracic kyphosis, and lumbar lordosis. Age, the bone mineral density score, and the presence of vacuum signs were not significantly different. However, there was a trend indicating that the degree of correction was higher in the group with new bone formation. The better radiographic parameters observed in this group may have led to faster recovery and better clinical outcomes. Park et al. reported that the mean restoration rates at the anterior and middle column immediately after vertebroplasty were significantly larger in patients with vacuum clefts who had undergone vertebroplasty [31]. A similar re-collapse rate was reported among groups with and without vacuum clefts. It was thus inferred that vertebroplasty in collapsed vertebrae with vacuum clefts might lead to better restoration of vertebral height and the original alignment and thus provide better stability for new bone formation. However, it was also reported that significant re-collapse was observed in patients with vacuum clefts undergoing vertebroplasty, with predictors including preoperative severe kyphotic deformity (a cutoff value of 12.5°), a solid lump cement distribution pattern, and a larger reduction angle (a cutoff value of 8.3°) [32]. In our study, there were 11 cases (7%) who underwent VP of the same vertebra due to re-collapse in the 3-year follow-ups. Analyzing the cases with re-collapse, we found that the median preoperative wedge angle was 18.03°, and the median reduced wedge angle was 6.49°. This was similar to the findings mentioned in the article above.

### 4.4. Limitations

The present study had several limitations. First, it was a retrospective study without a control group, and comparisons with conservative treatments and other treatment modalities were not conducted. Secondly, the postoperative radiographs varied in terms of the timeline between individuals for different follow-up strategies among surgeons as well as the compliance of patients. Thus, the use of plain films based on intervals may have affected the results. Because the primary outcome was the longitudinal measurements, generalized estimating equations (GEE) were used to determine the correlations between multiple results. Thirdly, in the present study, only changes in the radiographic parameters were investigated rather than the clinical parameters. No visual analogue scale (VAS) or other functional scores were recorded, so the relationship between the radiographic and clinical outcomes after PVP could not be correlated in the present study. Further studies on the correlations between clinical outcomes and image findings could be conducted to determine clinical relevance.

## 5. Conclusions

In the present retrospective study, we concluded that PVP significantly restored the wedge angles and compression ratios up to one postoperative year and that new bone formation was noted on plain radiographs, which increased over time. The restoration of vertebral parameters may contribute to new bone formation. Comparing to the non-operative control group, new bone formation was statistically higher in the PVP group. The non-operative control group had higher wedge angles compared with the PVP group. Further studies are needed to correlate these findings with clinical parameters.

## Figures and Tables

**Table 1:** Demographic data of included subjects.

			Q1–Q3
**Gender**	**Male**	38 (24.2)	
	**Female**	119 (75.8)	
**Age**		75.2 (7.95)	71, 76, 81
**T-score**		−2.76 (1.08)	−3.6, −2.9, −1.9
**Vacuum sign**	**+**	115 (73.2)	
	**−**	42 (26.8)	
**Amount of cement (mL)**		3.72 (1.94)	2.5, 3.3, 4.9

Gender and vacuum sign data are expressed as a number (percentage), and age, T-score, and amount of cement amount are expressed as a mean (standard deviation). First to third quartiles were expressed in Q1–Q3.

**Table 2:** Comparison of the groups with and without new bone formation after vertebroplasty. (* adjusted *p* value < 0.05).

Variables	Month	Callus (+)	Callus (−)	*p*-Value	Adj. p
**Age**	6	79	75	0.31	0.62
	12	79	75	0.07	0.28
	24	78.5	75	0.17	0.51
	36	77	76	0.76	0.76
**T-score**	6	−2.75	−2.80	0.69	0.84
	12	−2.90	−2.60	0.14	0.56
	24	−2.90	−2.60	0.23	0.69
	36	−2.90	−2.60	0.42	0.84
**Vacuum (−)** **Vacuum (+)**	6	4 (13%)	27 (87%)	0.16	0.48
	6	21 (28%)	54 (72%)		
	12	9 (29%)	22 (71%)	0.22	0.48
	12	33 (44%)	42 (56%)		
	24	11 (35%)	20 (65%)	0.07	0.28
	24	43 (57%)	32 (43%)		
	36	16 (52%)	15 (48%)	0.40	0.48
	36	47 (63%)	28 (37%)		
**Amount of Cement**	6	4.15	3.20	0.01	0.04 *
	12	4.00	3.20	0.04	0.12
	24	3.75	3.30	0.22	0.44
	36	3.50	3.40	0.45	0.45
**Wedge angle**	6	15.56	16.17	0.66	>0.99
	12	15.41	16.49	0.69	>0.99
	24	15.87	16.12	0.67	>0.99
	36	14.55	16.66	0.99	>0.99
**Anterior compression ratio**	6	0.47	0.49	0.57	>0.99
	12	0.47	0.49	0.94	>0.99
	24	0.47	0.50	0.97	>0.99
	36	0.46	0.51	0.52	>0.99
**Middle compression ratio**	6	0.58	0.54	0.28	>0.99
	12	0.56	0.54	0.43	>0.99
	24	0.55	0.53	0.55	>0.99
	36	0.54	0.54	0.81	>0.99
**Thoracic kyphotic angle**	6	48.54	39.50	0.84	>0.99
	12	38.82	40.18	0.77	>0.99
	24	37.57	44.88	0.46	>0.99
	36	37.69	44.88	0.76	>0.99
**Lumbar lordotic angle**	6	31.09	36.59	0.96	>0.99
	12	31.83	37.79	0.56	>0.99
	24	30.35	38.93	0.22	0.66
	36	30.34	39.77	0.16	0.64
**Wedge angle correction**	6	−7.38	−3.76	0.01	0.04 *
	12	−7.04	−3.52	0.03	0.09
	24	−6.63	−3.4	0.03	0.09
	36	−6.15	−3.89	0.26	0.26
**ACR correction**	6	−0.19	−0.09	0.07	0.14
	12	−0.17	−0.07	0.02	0.06
	24	−0.18	−0.06	0.01	0.04 *
	36	−0.14	−0.07	0.09	0.14
**MCR correction**	6	−0.15	−0.08	0.18	0.54
	12	−0.13	−0.07	0.12	0.48
	24	−0.12	−0.07	0.24	0.54
	36	−0.11	−0.06	0.28	0.54

**Table 3:** Univariate and Multivariate analysis of the factors contributing to new bone formation.

Univariate Analysis		
Variables	OR (95%)	*p*-Value
**Age**	1.03 (0.98, 1.07)	0.241
**T-score**	0.88 (0.65, 1.2)	0.430
**Amount of cement**	1.19 (1, 1.42)	0.052
**Vacuum**	1.82 (0.92, 3.61)	0.084
**Pre-op wedge angle**	1.01 (0.97, 1.05)	0.699
**Pre-op ACR**	1.13 (0.27, 4.74)	0.864
**Pre-op MCR**	2.67 (0.37, 19.36)	0.331
**Pre-op thoracic kyphosis**	1 (0.97, 1.04)	0.893
**Pre-op lumbar lordosis**	0.98 (0.96, 1.01)	0.249
**Wedge angle correction**	1.05 (0.99, 1.11)	0.105
**ACR correction**	10.7 (0.87, 131.46)	0.064
**MCR correction**	4.8 (0.27, 84.14)	0.283
**Thoracic kyphosis correction**	1.2 (0.98, 1.48)	0.073
**Lumbar lordosis correction**	0.91 (0.85, 0.97)	0.004
**Multivariate Analysis**		
**Variables**	OR (95%)	*p*-value
**Age**	1.02 (−0.03, 0.08)	0.44
**ACR correction**	15.98 (0, 5.54)	0.05

OR: Odds ratio.
